# Development of the Korean Facial Emotion Stimuli: Korea University Facial Expression Collection 2nd Edition

**DOI:** 10.3389/fpsyg.2017.00769

**Published:** 2017-05-12

**Authors:** Sun-Min Kim, Ye-Jin Kwon, Soo-Yun Jung, Min-Ji Kim, Yang Seok Cho, Hyun Taek Kim, Ki-Chun Nam, Hackjin Kim, Kee-Hong Choi, June-Seek Choi

**Affiliations:** Department of Psychology, Korea UniversitySeoul, South Korea

**Keywords:** facial emotion stimuli, database, facial action coding system, universal emotions

## Abstract

**Background:** Developing valid emotional facial stimuli for specific ethnicities creates ample opportunities to investigate both the nature of emotional facial information processing in general and clinical populations as well as the underlying mechanisms of facial emotion processing within and across cultures. Given that most entries in emotional facial stimuli databases were developed with western samples, and given that very few of the eastern emotional facial stimuli sets were based strictly on the Ekman’s Facial Action Coding System, developing valid emotional facial stimuli of eastern samples remains a high priority.

**Aims:** To develop and examine the psychometric properties of six basic emotional facial stimuli recruiting professional Korean actors and actresses based on the Ekman’s Facial Action Coding System for the Korea University Facial Expression Collection-Second Edition (KUFEC-II).

**Materials And Methods:** Stimulus selection was done in two phases. First, researchers evaluated the clarity and intensity of each stimulus developed based on the Facial Action Coding System. Second, researchers selected a total of 399 stimuli from a total of 57 actors and actresses, which were then rated on accuracy, intensity, valence, and arousal by 75 independent raters.

**Conclusion:** The hit rates between the targeted and rated expressions of the KUFEC-II were all above 80%, except for fear (50%) and disgust (63%). The KUFEC-II appears to be a valid emotional facial stimuli database, providing the largest set of emotional facial stimuli. The mean intensity score was 5.63 (out of 7), suggesting that the stimuli delivered the targeted emotions with great intensity. All positive expressions were rated as having a high positive valence, whereas all negative expressions were rated as having a high negative valence. The KUFEC II is expected to be widely used in various psychological studies on emotional facial expression. KUFEC-II stimuli can be obtained through contacting the corresponding authors.

## Introduction

The processing of human facial emotions has enjoyed considerable attention from diverse disciplines, ranging from basic science (McCarthy et al., 1997; Adolphs et al., 2005; Olofsson et al., 2008) to applied science (Russell, 1994; Öhman et al., 2001; Leppänen, 2006; Harms et al., 2010; Li et al., 2010). Human facial emotions function as a medium for interpersonal and social communication, therefore the ability to identify and discriminate others’ facial emotions is essential for effectively interacting and connecting with others in various social contexts (Russell, 2003). In order to examine the nature of human facial information processing, researchers have investigated populations with a reduced capacity to process human facial information, such as patients with traumatic brain injury (e.g., amygdala) (Barod et al., 1986; Adolphs et al., 1999), schizophrenia (Mandal et al., 1998; Kohler et al., 2003), bipolar disorder (Getz et al., 2003; Rocca et al., 2009), and autism spectrum disorder (Harms et al., 2010). Facial information processing has also been investigated in many areas within the typically developed population, from the developmental processes (e.g., Herba and Phillips, 2004; Durand et al., 2007) to the role of cognitive functions (e.g., Carretié et al., 2001; Pessoa et al., 2002; D’Argembeau and Van der Linden, 2007).

The universality of some human facial expressions conveying basic emotion has been proposed (Ekman et al., 1969). Ekman and Friesen (1978), as well as Izard and Weiss (1979), have pioneered and contributed to research on emotional human facial expressions, and to their coding systems, which assume that basic emotions are universally and innately recognizable across cultures. The universality of basic facial emotions has been recently re-evaluated, with reports of some disparities across cultures in the mental representations of the six basic emotions (i.e., easterners vs. westerners) (Jack et al., 2012) and the potential for culture to shape basic emotion expressions and perceptions, especially for emotions other than happiness (Crivelli et al., 2016). That is, cultural and ethnic differences may modulate the expressions and perception of the six basic emotions. For instance, people recognize familiar, in-group faces with greater sensitivity than unfamiliar, out-group faces (Ekman et al., 1987; Feingold, 1914). In addition, Chinese people living in China and the United States, Chinese Americans, and non-Asian Americans showed greater accuracy and took a shorter time when judging the facial emotions of people of the same race (Elfenbein and Ambady, 2003). Jack et al. (2012) insisted that these discrepancies reflect the role of nurture in biologically determined behaviors, such as the expression and perception of basic emotions.

Therefore, to investigate the role of culture on the six basic emotion expressions and perception, the need and interest in the development of culture-specific facial expression databases is growing (Shih et al., 2008). Indeed, Lyons et al. (1998) have developed a set of Japanese emotional expression stimuli, and Yin et al. (2006) have developed a set of Chinese stimuli using 3D components. We have previously developed a Korean Facial Expression stimuli set (KUFEC I) using 46 non-professional actors, based on the Ekman coding system (Kim et al., 2011). Our stimuli set together with other Korean expression sets, developed by other research groups in Korea (Lee et al., 2008, 2013), comprise a database that has facilitated a wide range of psychological and clinical studies of emotion perception in Korea (e.g., Lee et al., 2010).

However, questions about the existing KUFEC stimuli arose. First, these questions mainly surrounded the accuracy of the facial expressions based on Ekman’s facial action coding system (FACS; Ekman et al., 2002). Even though the KUFEC stimuli were developed based on the FACS, there were no training guidelines for actors, resulting in deviations in some stimuli from the FACS. Second, the existence of confounding variables (e.g., varying light, makeup, hairstyles, wearing glasses, etc.) may limit the use of the database in rigorous behavioral research (Saegusa et al., 2015; Tagai et al., 2016). For instance, actors for the KUFEC had different levels of makeup. Since facial emotions expressed by faces wearing heavy makeup are more likely to be misinterpreted (Tagai et al., 2016), makeup should be controlled or erased while creating the stimuli.

These issues could result in difficulties when interpreting study results. For instance, even though it has been noted that fearful Korean facial expressions have markedly low consensus ratings (Bahk et al., 2015), it is unclear whether this is the result of issues (e.g., deviations from the FACS) with stimuli, or with characteristics specific to Koreans (e.g., reduced sensitivity to fearful expressions).

For the abovementioned reasons, we have revised the previously developed Korea University Facial Expression Collection (KUFEC-I; Kim et al., 2011)—the most widely used Korean facial emotion database. The Korea University Facial Expression Collection version 2 (KUFEC-II) includes images of 28 male and 29 female professional actors who were trained to express six basic emotions strictly per Ekman’s FACS described in Ekman and Friesen (2003, pp. 28, 192). For the study of the asymmetric aspects of facial emotion perception (Borod and Caron, 1980; Borod et al., 1988; Nicholls et al., 2002), each facial expression was taken from three angles (i.e., 90, 45, and 135°). Confounding variables that could draw observers’ attention, such as makeup and varying lighting, were carefully controlled. Therefore, the KUFEC-II stimuli can be used to investigate various unsolved questions and debates regarding the six basic emotions.

The development of the KUFEC-II was conducted in the following three phases: (1) preparation for stimuli production, (2) producing facial emotion stimuli as static images, and (3) experts’ selecting the best stimuli. To evaluate the psychometric properties of the KUFEC-II, the final set stimuli set was rated by 75 participants for (A) valence, (B) arousal, (C) type, and (D) intensity.

### Database Development

The process of developing the KUFEC-II was carried out through three phases: preparation for production, creating emotional image stimuli, and selecting the best stimuli set.

#### Preparation/Apparatus

**FIGURE 1:**
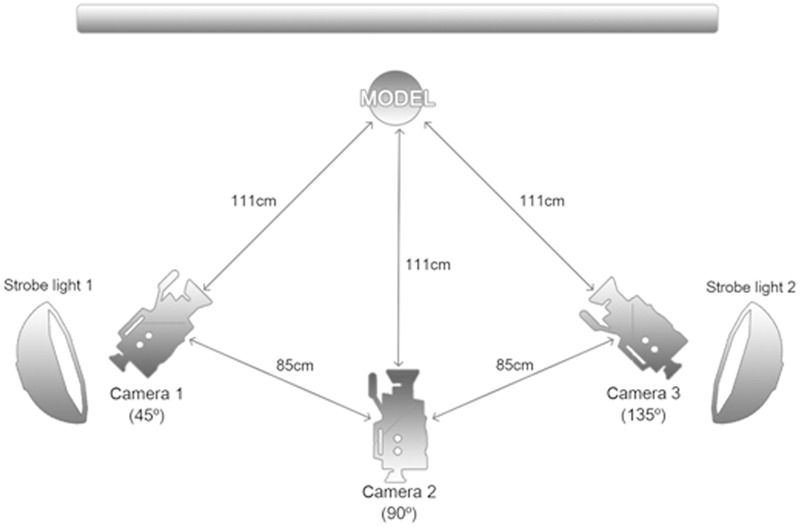
**Settings for Image Taking**.

Three digital single lens reflex (DSLR) cameras were used to create high-resolution emotional stimuli. The cameras were positioned in three places around the actors: in front and to left and right at 45° angles (**Figure 1**). All cameras captured photographs with a linked wireless release button to simultaneously take pictures. The position of the actor’s nose was kept at the center of all images, ensuring all stimuli had a similar composition. A gray background was used to minimize the influence of background distractors. Furthermore, we controlled extraneous variables related to the actors, such as clothes, hairstyles, hair color, various accessories, and makeup. All actors wore the same clothing and fixed their hair to show their hairline and ears. Accessories and makeup were removed before image production, and actors whose hair was dyed dramatically were excluded.

#### Image Production

All actors depicted the seven facial expressions: neutral, happiness, sadness, surprise, fear, anger, and disgust. Each emotion was captured multiple times to give actors training time for best results.

**FIGURE 2:**
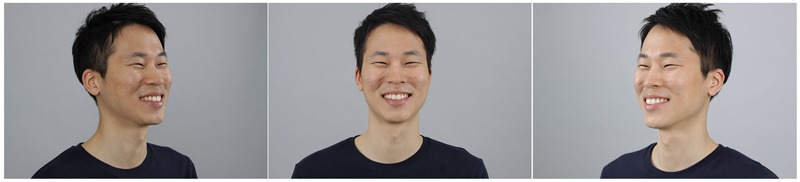
**Example of the images captured by three digital cameras (i.e., happiness)**.

Expressionless (neutral) faces were posed first as not to be influenced by any emotions. After capturing neutral faces, the FACS was introduced to the actors and they were coached by the authors based on Ekman’s FACS described in Ekman and Friesen (2003). Detailed directions, and information about the emotions to be portrayed, were given while photographing emotional expressions. The six-basic emotional facial expressions were photographed in a specific order (happiness, sadness, surprise, fear, anger, and disgust). Actors were asked to make their expressions as intense as possible to clearly depict the target emotion. The expressions were photographed at three angles simultaneously (**Figure 2**).

#### Selecting the Best Stimuli

The purpose of stimuli selection was to identify the images best representing the six emotions from all the actors. During the first step, all the pictures taken of actors were included for an initial evaluation; there were 68 actors (32 males and 36 females). Approximately 300 pictures were taken of each actor through multiple takes, and approximately 20,400 pictures were created and included for evaluation. The initial evaluation resulted in the exclusion of stimuli that contained photographic defects (e.g., de-focusing, low level of light, etc.), which resulted in the exclusion of pictures of 11 actors and the inclusion of approximately 17,100 pictures of 57 actors.

During the second step, for content validity of the stimuli, FACS-trained raters (Ekman et al., 2002) evaluated and selected the best emotion-depicting stimuli for each actor. The selection was made by a consensus among FACS-trained raters. Only one stimulus that was considered best was retained for each emotional category from each actor; thus, 399 pictures were retained (7 emotional categories × 57 actors). The raters conducted evaluations by judging (1) whether the corresponding emotions were properly expressed in each face (i.e., purity), and (2) whether the corresponding emotions were clearly expressed in each face (i.e., intensity). The manual for rating facial expression based on FACS (Ekman et al., 2002) was used in this step to minimize raters’ subjective judgments. The manual details the appropriate muscular movement around eyes, nose, and mouth. It also contains the features of mixed emotions, to help raters evaluate the clarity of facial expressions.

**FIGURE 3:**
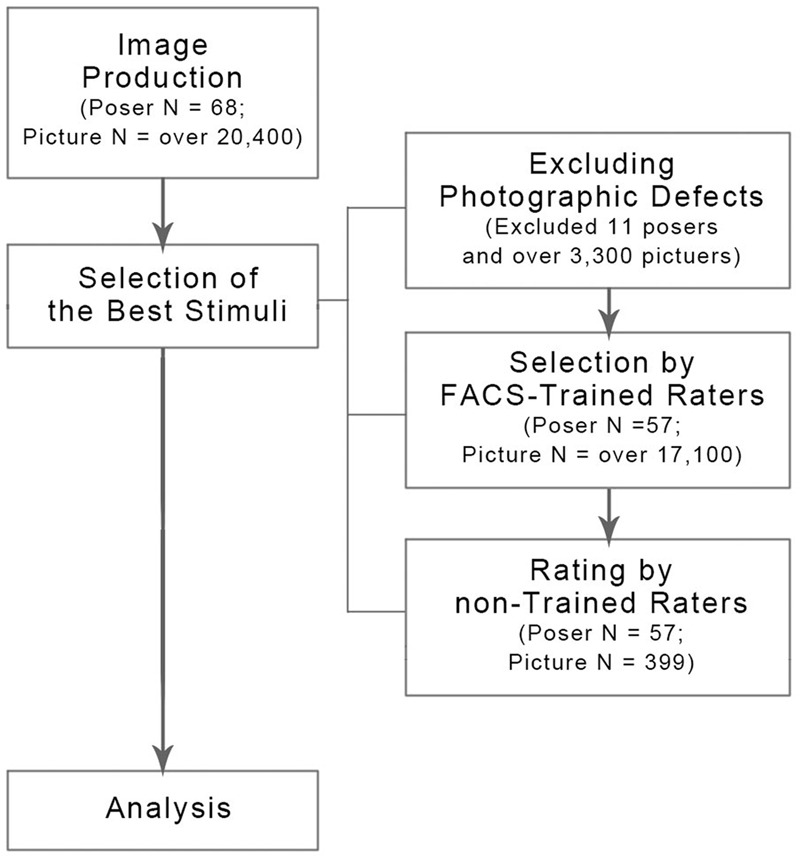
**Flow-chart of the development and validation of the KUFEC-II**.

During the third step, non-trained benign raters who were not familiar with the FACS evaluated the 399 pictures across four dimensions (i.e., valence, arousal, type, and intensity). A total of 399 stimuli were selected for the KUFEC-II. The final set contained seven images from each of the 57 actors. The process of producing and selecting stimuli for the database is presented in **Figure 3**.

## Materials and Methods

### Participants

The number of participants required to obtain significant results was analyzed a priori. In a previous study, the effect size for correctly identifying a targeted emotion (“hit rate”) out of eight type dimensions in eight models in the Radboud Faces Database (RaFD) was η^2^ = 0.47 (Langner et al., 2010). In addition, in a previous study, mean differences in accuracy between Western and Eastern models’ fearful facial expressions were large (*t* = 3.60, *df* = 45) (Bahk et al., 2015). Given the large effect sizes observed in previous studies, we decided that 54 participants should provide sufficient power for 80% power assuming a 5% significance level and a two-sided test.

Ninety-seven participants who voluntarily applied and agreed to participate were recruited. Seven volunteers were unable to get in touch after first contact, and 10 were ruled out due to the presence of past and present neuropsychological problems, including depression, suicidal ideation, hypomanic episodes, anxiety, obsessive thoughts, bulimia, or current alcohol use. The volunteers with past and present neuropsychological problems were excluded since previous studies have reported emotion perception biases in individuals with mental disorders (Salters-Pedneault et al., 2006; Harrison et al., 2009; Kohler et al., 2011).

Excluding five outliers in hit rate (correctly identifying the target emotion type in the stimuli), 75(*M* = 39, *F* = 36) raters were included in the final analysis. Data from five raters were excluded because their hit rates were lower than the criterion interquartile range (IQR) [i.e., first quartile (Q1) - 1.5 × IQR]. The raters’ age ranged from 19 to 69, and most were in their twenties (*M* = 26.17, *SD* = 5.69). The mean age for males was 26.79(*SD* = 7.76) and mean age for females was 25.51(*SD* = 1.76); the difference between genders was non-significant in terms of hit rate (*p* = 0.05). This study was carried out in accordance with the recommendations of the local Institutional Review Board with written informed consent from all subjects. All subjects gave written informed consent in accordance with the Declaration of Helsinki.

### Procedure

Before conducting the experiment, all participants were interviewed by the researchers to screen for past or present neuropsychiatric disorders using the Mini International Neuropsychiatric Interview (Sheehan et al., 1998).

Prior to rating facial emotion stimuli, participants were asked to report their affective states during prior week using the Positive Affect and Negative Affect Schedule (Watson et al., 1988; Lee et al., 2003). The scale was used to measure and control the potential influence of individuals’ affective states when rating the facial emotion stimuli.

For rating the KUFEC-II stimuli, participants were given instructions regarding the four dimensions to be rated (i.e., valence, intensity, arousal, and emotion type). Given that it required approximately 90–120 min to complete, a 10-min break between the first-half and second-half was provided to help participants maintain proper concentration and reduce fatigue. This study was carried out in accordance with the recommendations of Korea University Institutional Review Boards with written informed consent from all subjects. All subjects gave written informed consent in accordance with the Declaration of Helsinki. The protocol was approved by the Korea University Institutional Review Boards.

### Measures

#### Mini International Neuropsychiatric Interview (MINI)

Mini International Neuropsychiatric Interview is structured interview for assessing psychiatric disorders based on the fourth edition of the diagnostic and statistical manual of mental disorders (DSM-IV), and the International classification of Disease, 10th revision (ICD-10). MINI’s kappa value was between 0.51 and 0.76 for the original version (Sheehan et al., 1998) and 0.62 to 0.81 for the Korean version (Yoo et al., 2006).

#### Positive Affect and Negative Affect Schedule (PANAS)

The Positive Affect and Negative Affect Schedule was developed to assess an individual’s affective state (Watson et al., 1988). Internal consistency reliability for the original version of the scale was 0.89, with both positive and negative subscales scoring 0.85. The internal consistency reliability of the Korean version of the PANAS was 0.88, with each subscale scoring 0.87 (Lee et al., 2003). In the current study, the positive scale scored 0.90 while the negative scale scored 0.95.

#### Rating the KUFEC-II Stimuli

The KUFEC-II stimuli were presented on a computer screen using E-prime 2.0. Each stimulus was shown in a 19-inch monitor with a resolution of 1280 × 1024. Each image was presented at the center of a white background in a random order. Only frontal facial stimuli (90°) were included for rating. A total of 399 stimuli (a total of 57 actors’ facial emotions) of seven types of emotional expression were evaluated on a 7-point Likert scale. For valence dimensions, the participants were required to determine whether the stimuli portrayed a positive or negative expression, and how clear they were. Anchors are on a 7-point Likert scale (i.e., ranging from 1 indicating an extremely negative expression to 7 an extremely positive expression). For arousal dimensions, the instruction was worded as “please assess how you are physiological or emotionally aroused when looking at the expression.” Anchors are on a 7-point Likert scale (i.e., ranging from 1 indicating no arousal response to 7 an intensely aroused state). We also provided additional examples (i.e., “When someone sees a person who is very scared, the person might feel terrified or have physiological responses, such as a racing heart. The emotional and/or physiological responses reflect arousal in your body”). For type dimensions, the participants were asked to choose the best emotional word out of seven (i.e., happiness, sadness, surprise, fear, anger, disgust, and neutral) that most clearly represented the facial expression in the stimulus. We also provided examples for each emotion (i.e., “We feel surprised when we encounter unexpected situations”). For intensity dimensions, the participants were asked to rate how strong the emotion is depicted in each stimulus. Anchors are on a 7-point Likert scale (i.e., ranging from 1 indicating very weak to 7 very strong). The images were shown at the center of white background, and presentation order was randomized.

## Results

### Reliability of Stimuli

The internal consistencies of KUFEC-II stimuli were evaluated first. The intra-class correlation coefficients (ICCs) were 0.97, 1.00, 0.99, and 0.93 for accuracy, valence, arousal and intensity, respectively.

### Hit Rates

The average hit rate of the KUFEC-II stimuli (percentage of times images were rated to reflect the target emotion) was 81% (*SD* = 22.42). The hit rate is the indicator for “purity,” as it reflects the consensus rate of recognizing the stimuli as certain emotion, rather than other emotions (**Table 1**). The hit rate for each emotion was as follows: happiness 97% (*SD* = 1.66), sadness 84% (*SD* = 18.88), surprise 93% (*SD* = 4.39), fear 50% (*SD* = 16.32), anger 87% (*SD* = 13.87), disgust 63% (*SD* = 18.32), and neutral 93% (*SD* = 10.70). A one-way analysis of variation (ANOVA) was performed to test differences in hit rates between emotions. There were significant differences in hit rates between emotions, *F*(6,398) = 115.94, *p* < 0.001, η^2^ = 0.64. Scheffe *post hoc* tests showed that fearful and disgust faces had significantly lower hit rates than the other emotions (*p*s < 0.001; fearful vs. happy, *p* < 0.001, cohens *d* = 4.59; fearful vs. angry, *p* < 0.001, cohen’s *d* = 2.79; fearful vs. neutral, *p* < 0.001, cohen’s *d* = 3.44; fearful vs. sad, *p* < 0.001, cohen’s *d* = 2.30; fearful vs. surprise, *p* < 0.001, cohen’s *d* = 4.09; fearful vs. disgust, *p* < 0.001, cohen’s *d* = 1.09; disgust vs. happy, *p* < 0.001, cohen’s *d* = 2.64; disgust vs. angry, *p* < 0.001, cohen’s *d* = 1.44; disgust vs. fearful, *p* < 0.001, cohen’s *d* = 1.09; disgust vs. neutral, *p* < 0.001, cohen’s *d* = 1.90; disgust vs. sad, *p* < 0.001, cohen’s *d* = 1.17; disgust vs. surprise, *p* < 0.001, cohen’s *d* = 2.25).

**Table 1:** Percentage of chosen emotions per intended emotional expression (%).

	Happiness	Sadness	Surprise	Fear	Anger	Disgust	Neutral
Happiness	97.11	0.45	0.49	0.33	0.68	0.61	0.33
Sadness	0.26	84.38	1.17	1.12	3.35	4.50	5.22
Surprise	0.70	1.26	92.72	3.23	0.52	0.63	0.94
Fear	1.05	5.07	26.85	49.76	0.00	16.52	0.75
Anger	0.26	2.81	0.35	1.36	87.33	7.03	0.87
Disgust	0.45	8.81	1.34	2.16	23.58	63.46	0.21
Neutral	1.49	2.15	0.71	0.52	2.10	0.54	92.50

**FIGURE 4:**
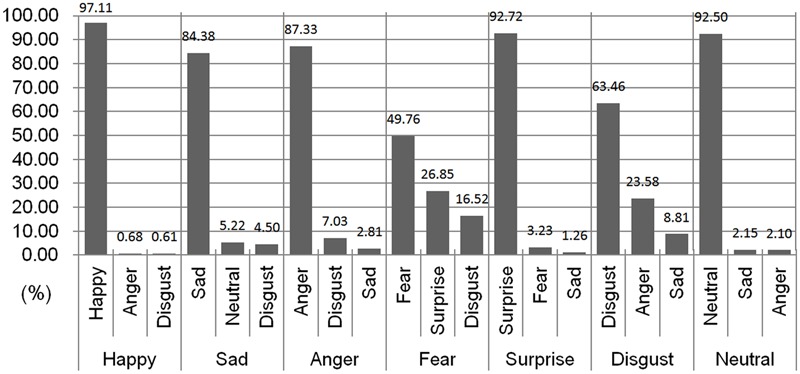
**Top 3 emotion types rated for each target emotion**.

Top 3 emotion types rated for each target emotion of six basic emotions and neutral faces are shown in **Figure 4**. The incorrect responses given most frequently and second most frequently for each emotion are presented with hit rates. Happiness, which had the highest hit rates, was mistaken for another emotion at a rate of less than 1%. Sad and neutral, anger and disgust, and fear and surprise were most frequently mislabeled and confused with one another. It is noteworthy that over 20% of participants mistook fear as surprise (26.85%) and disgust as anger (23.58%).

### Intensity

Mean intensity, valence, and arousal levels were calculated for each emotion (**Table 2**). There were significant differences between emotions in intensity (*p* < 0.001, η^2^ = 0.25). Results from the one-way ANOVA and Scheffe *post hoc* tests revealed that happy facial expressions were perceived to be more intense than other emotional expressions (*p*s < 0.05; vs. sad, *p* < 0.001, cohen’s *d* = 2.01; vs. fear, *p* < 0.001, cohen’s *d* = 1.36; vs. anger, *p* < 0.001, cohen’s *d* = 1.43; vs. surprise, *p* = 0.001, cohen’s *d* = 1.06; vs. disgust, *p* < 0.001, cohen’s *d* = 1.63). On the other hand, sad expressions were rated as less intense than fear, surprise, and neutral (*p*s < 0.05; vs. fear, *p* = 0.019, cohen’s *d* = 0.61; vs. surprise, *p* < 0.001, cohen’s *d* = 0.97; vs. neutral, *p* = 0.002, cohen’s *d* = 0.95).

**Table 2:** Mean scores (M) and Standard deviations (SD) for valence, arousal, and intensity level (*N* = 75).

	Valence		Arousal		Intensity	
	*M*	*SD*	*M*	*SD*	*M*	*SD*
Happy	6.27	0.22	4.21	0.25	6.18	0.30
Anger	2.00	0.35	4.53	0.47	5.52	0.58
Fear	2.25	0.40	4.73	0.40	5.61	0.52
Neutral	3.88	0.18	2.46	0.18	5.67	0.18
Sad	2.28	0.40	3.91	0.51	5.27	0.57
Surprise	3.59	0.19	4.55	0.44	5.77	0.45
Disgust	1.91	0.24	4.61	0.32	5.58	0.43

*A 7-point Likert scale was used (1–7), with higher scores reflecting greater positive affect, higher arousal levels, and stronger expressions.*

### Valence and Arousal

Mean valence and arousal levels were calculated, and a one-way analysis of variance (ANOVA) was conducted to see whether any differences existed between emotional categories in valence and arousal. There were significant differences between emotions in both valence and arousal (*p*s < 0.001; valence, *p* < 0.001, η^2^ = 0.96; arousal, *p* < 0.001, η^2^ = 0.79)

Happy facial expressions were perceived as more positive than other emotions (*p*s < 0.001; vs. sad, *p* < 0.001, cohen’s *d* = 12.27; vs. surprise, *p* < 0.001, cohen’s *d* = 13.06; vs. fear, *p* < 0.001, cohen’s *d* = 12.45; vs. anger, *p* < 0.001, cohen’s *d* = 14.54; vs. disgust, *p* < 0.001, cohen’s *d* = 18.99; vs. neutral, *p* < 0.001, cohen’s *d* = 11.77) (**Table 2**). Angry facial expressions were identified as more negative than other emotions (*p*s < 0.05; vs. happy, *p* < 0.001, cohen’s *d* = 14.54; vs. fear, *p* = 0.004, cohen’s *d* = 0.65; vs. neutral, *p* < 0.001, cohen’s *d* = 6.71; vs. sad, *p* < 0.001, cohen’s *d* = 0.73; vs. surprise, *p* < 0.001, cohen’s *d* = 5.64), except for disgust (*p* = 0.85). Neutral and surprised facial expressions were recognized as more negative than happy ones, and as more positive than the others (*p*s < 0.001; neutral vs. happy, *p* < 0.001, cohen’s *d* = 11.77; neutral vs. anger, *p* < 0.001, cohen’s *d* = 6.71; neutral vs. fear, *p* < 0.001, cohen’s *d* = 5.26; neutral vs. sad, *p* < 0.001, cohen’s *d* = 5.11; neutral vs. surprise, *p* < 0.001, cohen’s *d* = 1.56; neutral vs. disgust, *p* < 0.001, cohen’s *d* = 9.27; surprise vs. happy, *p* < 0.001, cohen’s *d* = 13.06; surprise vs. anger, *p* < 0.001, cohen’s *d* = 5.64; surprise vs. fear, *p* < 0.001, cohen’s *d* = 4.30; surprise vs. sad, *p* < 0.001, cohen’s *d* = 4.17; surprise vs. disgust, *p* < 0.001, cohen’s *d* = 7.83). Surprised expressions were perceived as being more negative than neutral ones (*p* < 0.001, cohen’s *d* = 1.56).

**FIGURE 5:**
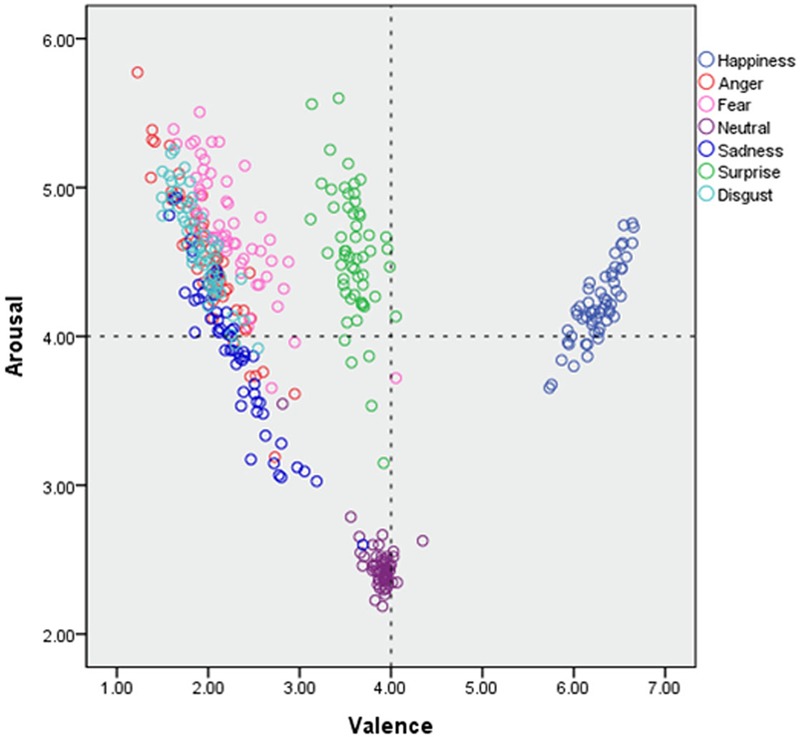
**Evaluation results of valence and arousal for KUFEC-II**.

For arousal, neutral stimuli were recognized as being less arousing than emotional stimuli (*p*s < 0.001; neutral vs. happy, *p* < 0.001, cohen’s *d* = 7.95; neutral vs. anger, *p* < 0.001, cohen’s *d* = 5.77; neutral vs. fear, *p* < 0.001, cohen’s *d* = 7.22; neutral vs. sad, *p* < 0.001, cohen’s *d* = 3.79; neutral vs. surprise, *p* < 0.001, cohen’s *d* = 6.18; neutral vs. disgust, *p* < 0.001, cohen’s *d* = 8.18) (**Table 2**). Fear, surprise, anger, and disgust expressions were reported to arouse raters more than happy or sad expressions (*p*s < 0.05; fear vs. happy, *p* < 0.001, cohen’s *d* = 1.52; fear vs. sad, *p* < 0.001, cohen’s *d* = 1.77; surprise vs. happy, *p* = 0.002, cohen’s *d* = 0.93; surprise vs. sad, *p* < 0.001, cohen’s *d* = 1.33; anger vs. happy, *p* = 0.005, cohen’s *d* = 0/83; anger vs. sad, *p* < 0.001, cohen’s *d* = 1.25; disgust vs. happy, *p* < 0.001, cohen’s *d* = 1.38; disgust vs. sad, *p* < 0.001, cohen’s *d* = 1.64) (**Table 2** and **Figure 5**).

**Figure 5** shows the dimensional classifications of emotion by valence. As shown in **Figure 5**, in all emotional expressions, there were positive relationships between valence and arousal.

### Gender Effects

A repeated measures analysis of variance (ANOVA) was conducted to test the effects of gender on stimuli evaluation in terms of hit rate. The effect of gender on precise perception of emotion (hit rate for each emotional category) was significant for both raters and actors (*p* < 0.001, η^2^ = 0.05 for raters; *p* < 0.001, η^2^ = 0.05 for actors). Female actors were rated as more precisely, and female raters scored higher hit rate than males. However, there were no significant interactions between raters’ and actors’ gender (*p* = 0.88), which means that there was no significant effect on recognizing emotions of same or opposite gender. The results are consistent with the previous studies, showing females are more effective than males in recognizing and expressing emotions (Drag and Shaw, 1967; Buck et al., 1974; Rotter and Rotter, 1988).

### Effects of Emotional State

In addition, to evaluate whether ratings would be influenced by each rater’s emotional state, correlations between emotional state measured by the PANAS and ratings on KUFEC-II stimuli for four dimensions (i.e., accuracy, valence, arousal, and intensity) were calculated (Supplementary Table 1). The results show that there were no significant correlations (*p*s > 0.05; positive affect, *p* = 0.55; negative affect, *p* = 0.66), indicating that the ability to identify emotions from faces was not associated with participants’ emotional state during the past week.

## Discussion

In the current study, we developed a novel Korean facial expression database, the Korea University Facial Expression Collection 2nd edition (KUFEC-II), and evaluated its psychometric properties along the dimensions of emotion type, valence, arousal, and intensity, supporting their validity and inter-rater reliability. With these improved features, KUFEC-II is expected to provide accurate FACS-based Korean facial stimuli for psychological, clinical, and/or engineering research both within Korean culture and across cultures, together with previously developed facial expression databases.

Specifically, the overall hit rate between the targeted and rated emotional expressions for the KUFEC-II was 81%, which is comparable to other widely used databases, such as the Radboud Face Database (RaFD, 82%; Langner et al., 2010) and the Karolinska Directed Emotional Faces Database (KDEF, 72%; Goeleven et al., 2008) (Supplementary Table 2). The hit rate (45%) for fearful facial expressions on the KUFEC-II has improved considerably compared to that of the KUFEC (13%). However, it is still the lowest among other emotional expressions in the KUFEC-II. Even though potential errors during the development of fearful and disgust facial expressions might account for lower hit rates, previous studies have reported that hit rates for fear and disgust seem relatively lower than those for other emotional expressions across different databases, such as the KDEF (43 and 72% for fear and disgust, respectively) and the ChaeLee-E (49 and 69% for fear and disgust, respectively), but not the RaFD (81 and 81% for fear and disgust, respectively). Interestingly, Biehl et al. (1997) reported that Japanese raters had lower hit rates for fear (59%) and disgust (73%) than Caucasian raters (81 and 83 % for fear and disgust, respectively) when they evaluated the same Japanese facial expressions. Thus, it is also possible that Korean raters may be less sensitive to or may interpret fearful or disgust facial expressions differently than Caucasian raters, which should be investigated in a future study.

With regard to the low hit rates and overlap between some facial expression ratings observed in the current study, the low agreement rate for fearful and disgust faces is not uncommon in other sets of facial expressions (Gur et al., 2002; Goeleven et al., 2008; Lee et al., 2013), except for Langner et al. (2010). In particular, Ekman and Friesen (1986) mentioned that disgust and anger are often confused with one another, and Yrizarry et al. (1998) reported that Japanese and Caucasian raters perceive multiple emotions in each basic emotion. It is still unclear why this overlap has been found in multiple studies across cultures. It is speculated that the overlap observed between some facial emotions (e.g., disgust and anger) might be due to morphological overlaps between these emotions (Goeleven et al., 2008; Langner et al., 2010). Namely, some facial features involved in making a specific facial expression (e.g., disgust or anger) activate facial muscles also involved in other facial expressions (e.g., nose wrinkle, lowered brow). Another possibility is that there is overlap in the semantic construct level among different emotions (Wyer et al., 2015). Particularly in Korea, many surprising events are perceived as negative or aversive, whereas surprise was originally categorized as neutral in Ekman’s classification system. Therefore, Koreans might have negative semantic images when they express or perceive surprising facial expressions. Interestingly, 27% of the non-trained raters in the current study perceived fearful stimuli as surprise (Supplementary Table 3). It should be an interesting research topic to investigate in a future study whether overlap between facial expression ratings would be due to morphological uniqueness of Korean actors, or due to differences in semantic construct levels among emotions. We believe that this research question could be addressed by employing both a set of evoked facial emotion stimuli and FACS coded facial stimuli (e.g., KUFEC-II) being rated by both Korean and non-Korean raters.

High mean intensity score of 5.63 indicates that KUFEC-II stimuli express targeted facial emotions with strong intensity. For neutral stimuli, it is speculated that high intensity ratings reflect genuineness of the targeted neutral expressions.

All positive expressions showed high valence whereas all negative expression showed low valence, which is consistent with previous studies, except for surprise (Ekman and Friesen, 2003).

The valence results of surprised faces on evaluation can be explained by the possibility of negatively biased facial expressions used by the Korean actors. In fact, neutral expressions were also rated as slightly below the middle value of 4, showing a slightly negative valence (*M* = 3.88, midpoint at 4), when several studies in western cultures showed that neutral faces were rated as slightly positive (Langner et al., 2010; *M* = 3.0, midpoint at 3). This might be because Koreans are not used to making the courteous, positive, or surprising faces found in western culture (e.g., expression of “Really?”). Another explanation is based on perceiver bias. Surprise is an emotional response to unexpected event that precedes causal thinking (Stiensmeier-Pelster et al., 1995), and it is common to conceal positive affect toward unexpected joyful things (e.g., promotion, success in examination, etc.) in Korean culture, which naturally causes a tendency to express surprised feelings mostly when unexpected negative events happen. It is generally considered a virtue in Korea not to express feelings about pleasing personal events, especially when the event can cause others’ jealousy. Therefore, the valence judging system of average Koreans might be biased toward the negative. As mentioned above, a future study might address this interesting topic by employing both a set of evoked facial emotion stimuli and FACS coded facial stimuli (e.g., KUFEC-II) being rated by both Korean and non-Korean raters.

Several limitations should be noted. First, the age of raters represents a limitation in our study. Most of the raters were in their twenties and thirties. There are studies demonstrating the decline of emotional recognition ability due to aging and cognitive deterioration (Orgeta and Phillips, 2007; Ruffman et al., 2008). Therefore, the data reported in this article might be amplified by age, and our results cannot be generalized to other age groups. Still, our data characterize the responses of people with ideal capabilities. Second, since levels of attractiveness would influence on emotion recognition (Hosoda et al., 2003), in a future study, it should be investigated that ratings on dimensions of hit rates, valence, intensity and arousal would be varied by levels of attractiveness of actors in KUFEC-II.

The ratings on the four dimensions for each stimulus were conducted simultaneously with a fixed order (i.e., valence, arousal, type, and intensity). The experimental process was formulated to minimize the effect of emotional category on the valence and arousal evaluations, such that questions regarding emotion type were presented after those regarding valence and arousal. However, it is possible that ratings on one dimension (e.g., valence) may have influenced ratings on other dimensions (e.g., arousal). Even though “sadness” and “anger” are both negatively valenced, the non-trained raters evaluated anger as more arousing than sadness. Even though it is difficult to infer whether the valence evaluation systematically affected the arousal evaluation, the influence of order effects should be investigated in a future study.

In the current study, Korean facial expression stimuli for six basic emotions were developed and validated, but some interesting research questions remain for future work. Although this study did not find a significant correlation between the ability to identify emotions from faces and emotional state during the past week, previous studies suggest that emotional state has a possible influence on emotion perception (Niedenthal et al., 2000). Therefore, it seems meaningful to investigate in a future study whether emotion perception is influenced by various emotional state during shorter-term (i.e., right now or today) and longer-term period (i.e., past year or in general). Furthermore, as only static images were included in the KUFEC-II, developing dynamic clips of emotional expressions would be useful to increase the utility of the database and to allow for more diverse research.

The KUFEC-II provides facial expression stimuli for all the six basic emotions, carefully applied to Ekman’s facial action coding system. These fifty-seven sets of stimuli are expected to enable researchers to have more options to fit their own research themes. Additionally, the rigorous restriction of confounding variables makes the KUFEC-II available to experimental researchers requiring strict control. It can also be applicable to intervention research using facial expression or assessment processes.

## Author Contributions

K-HC and J-SC designed the study and supervised overall research processes including assessment, data management, subject recruitment, stimuli development and data analysis. S-MK participated in stimuli development, performed the literature research and statistical analyses, and wrote the first draft of the manuscript. Y-JK and S-YJ performed the literature research, conducted assessment and experiments. HTK, K-CN, and HJK consulted on the research design. Subsequent drafts of the manuscript were revised by all the authors. All authors contributed to and have approved the final manuscript.

## Conflict of Interest Statement

The authors declare that the research was conducted in the absence of any commercial or financial relationships that could be construed as a potential conflict of interest.

## References

[B1] Adolphs R. Gosselin F. Buchanan T. W. Tranel D. Schyns P. Damasio A. R. (2005). A mechanism for impaired fear recognition after amygdala damage. *Nature* 433 68–72. 10.1038/nature03086 15635411

[B2] Adolphs R. Tranel D. Hamann S. Young A. W. Calder A. J. Phelps E. A. (1999). Recognition of facial emotion in nine individuals with bilateral amygdala damage. *Neuropsychologia* 37 1111–1117. 10.1016/S0028-3932(99)00039-1 10509833

[B3] Bahk Y. C. Jang S. K. Lee J. Y. Choi K. H. (2015). Korean facial emotion recognition tasks for schizophrenia research. *Psychiatry Investig.* 12 235–241. 10.4306/pi.2015.12.2.235 PMC4390595 25866525

[B4] Barod J. C. Koff E. Perlman Lorch M. Nicholas M. (1986). The expression and perception of facial emotion in brain-damaged patients. *Neuropsychologia* 24 169–180. 10.1016/0028-3932(86)90050-3 3714022

[B5] Biehl M. Matsumoto D. Ekman P. Hearn V. Heider K. Kudoh T. (1997). Matsumoto and Ekman’s Japanese and Caucasian Facial Expressions of Emotion (JACFEE): reliability data and cross-national differences. *J. Nonverbal. Behav.* 21 3–21. 10.1023/A:1024902500935

[B6] Borod J. C. Caron H. S. (1980). Facedness and emotion related to lateral dominance, sex and expression type. *Neuropsychologia* 18 237–242. 10.1016/0028-3932(80)90070-6 7383316

[B7] Borod J. C. Kent J. Koff E. Martin C. Alpert M. (1988). Facial asymmetry while posing positive and negative emotions: support for the right hemisphere hypothesis. *Neuropsychologia* 26 759–764. 10.1016/0028-3932(88)90013-9 3211295

[B8] Buck R. Miller R. E. Caul W. F. (1974). Sex, personality, and physiological variables in the communication of affect via facial expression. *J. Pers. Soc. Psychol.* 30 587–596. 10.1037/h0037041 4455775

[B9] Carretié L. Mercado F. Tapia M. Hinojosa J. A. (2001). Emotion, attention, and the ‘negativity bias’, studied through event-related potentials. *Int. J. Psychophysiol.* 41 75–85. 10.1016/S0167-8760(00)00195-1 11239699

[B10] Crivelli C. Jarillo S. Russell J. A. Fernández-Dols J. M. (2016). Reading emotions from faces in two indigenous societies. *J. Exp. Psychol. Gen.* 145 830–843. 10.1037/xge0000172 27100308

[B11] D’Argembeau A. Van der Linden M. (2007). Facial expressions of emotion influence memory for facial identity in an automatic way. *Emotion* 7 507–515. 10.1037/1528-3542.7.3.507 17683207

[B12] Drag R. M. Shaw M. E. (1967). Factors influencing the communication of emotional intent by facial expressions. *Psychon. Sci.* 8 137–138. 10.3758/BF03331587

[B13] Durand K. Gallay M. Seigneuric A. Robichon F. Baudouin J. Y. (2007). The development of facial emotion recognition: the role of configural information. *J. Exp. Child Psychol.* 97 14–27. 10.1016/j.jecp.2006.12.001 17291524

[B14] Ekman P. Friesen W. V. (1978). *Manual of the Facial Action Coding System (FACS).* Palo Alto, CA: Consulting Psychologist Press.

[B15] Ekman P. Friesen W. V. (1986). A new pan-cultural facial expression of emotion. *Motivat. Emot.* 10 159–168. 10.1007/BF00992253

[B16] Ekman P. Friesen W. V. (2003). *Unmasking the Face: A Guide to Recognizing Emotions from Facial Clues.* Los Altos, CA: ISHK.

[B17] Ekman P. Friesen W. V. Hager J. (2002). *Emotional Facial Action Coding System. Manual and Investigators Guide.* CD-ROM. Salt Lake City, UT: Human Face.

[B18] Ekman P. Friesen W. V. Osullivan M. Chan A. Diacoyannitarlatzis I. Heider K. (1987). Universals and cultural-differences in the judgments of facial expressions of emotion. *J. Pers. Soc. Psychol.* 53 712–717. 10.1037/0022-3514.53.4.712 3681648

[B19] Ekman P. Sorenson E. R. Friesen W. V. (1969). Pan-cultural elements in facial displays of emotion. *Science* 164 86–88. 10.1126/science.164.3875.86 5773719

[B20] Elfenbein H. A. Ambady N. (2003). When familiarity breeds accuracy: cultural exposure and facial emotion recognition. *J. Pers. Soc. Psychol.* 85 276–290. 10.1037/0022-3514.85.2.276 12916570

[B21] Feingold G. A. (1914). The influence of environment on identification of persons and things. *J. Crim. Law Criminol.* 5 39–51. 10.2307/1133283

[B22] Getz G. E. Shear P. K. Strakowski S. M. (2003). Facial affect recognition deficits in bipolar disorder. *J. Int. Neuropsychol. Soc.* 9 623–632. 10.1017/S1355617703940021 12755174

[B23] Goeleven E. De Raedt R. Leyman L. Verschuere B. (2008). The Karolinska directed emotional faces: a validation study. *Cogn. Emot.* 22 1094–1118. 10.1080/02699930701626582

[B24] Gur R. C. Sara R. Hagendoorn M. Marom O. Hughett P. Macy L. (2002). A method for obtaining 3-dimensional facial expressions and its standardization for use in neurocognitive studies. *J. Neurosci. Methods* 115 137–143. 10.1016/S0165-0270(02)00006-7 11992665

[B25] Harms M. B. Martin A. Wallace G. L. (2010). Facial emotion recognition in Autism spectrum disorders: a review of behavioral and neuroimaging studies. *Neuropsychol. Rev.* 20 290–322. 10.1007/s11065-010-9138-6 20809200

[B26] Harrison A. Sullivan S. Tchanturia K. Treasure J. (2009). Emotion recognition and regulation in anorexia nervosa. *Clin. Psychol. Psychother.* 16 348–356. 10.1002/cpp.628 19517577

[B27] Herba C. Phillips M. (2004). Annotation: development of facial expression recognition from childhood to adolescence: behavioural and neurological perspectives. *J. Child Psychol. Psychiatry* 45 1185–1198. 10.1111/j.1469-7610.2004.00316.x 15335339

[B28] Hosoda M. Stone-romero E. F. Coats G. (2003). The effects of physical attractiveness on job-related outcomes: a meta-analysis of experimental studies. *Pers. Psychol.* 56 431–462. 10.1111/j.1744-6570.2003.tb00157.x

[B29] Izard C. E. Weiss M. (1979). *The Maximally Discriminative Facial Movement Coding System (MAX).* Newark, DE: University of Delaware.

[B30] Jack R. E. Garrod O. G. Yu H. Caldara R. Schyns P. G. (2012). Facial expressions of emotion are not culturally universal. *Proc. Natl. Acad. Sci. U.S.A.* 109 7241–7244. 10.1073/pnas.1200155109 22509011 PMC3358835

[B31] Kim M. W. Choi J. S. Cho Y. S. (2011). The Korea university facial expression collection (KUFEC) and semantic differential ratings of emotion. [The Korea university facial expression collection (KUFEC) and semantic differential ratings of emotion]. *J. Exp. Psychol.* 30 1189–1211.

[B32] Kohler C. G. Hoffman L. J. Eastman L. B. Healey K. Moberg P. J. (2011). Facial emotion perception in depression and bipolar disorder: a quantitative review. *Psychiatry Res.* 188 303–309. 10.1016/j.psychres.2011.04.019 21601927

[B33] Kohler C. G. Turner T. H. Bilker W. B. Brensinger C. M. Siegel S. J. Kanes S. J. (2003). Facial emotion recognition in schizophrenia: intensity effects and error pattern. *Am*. *J. Psychiatry* 160 1768–1774. 10.1176/appi.ajp.160.10.1768 14514489

[B34] Langner O. Dotsch R. Bijlstra G. Wigboldus D. H. J. Hawk S. T. van Knippenberg A. (2010). Presentation and validation of the Radboud Faces Database. *Cogn. Emot.* 24 1377–1388. 10.1080/02699930903485076

[B35] Lee H. Park S. Kang B. Shin J. Lee J. Je H. (2008). “The POSTECH face database (PF07) and performance evaluation,” in *Proceedings of the 8th IEEE International Conference on Automatic Face and Gesture Recognition (FG 2008)* Amsterdam.

[B36] Lee H. H. Kim E. J. Lee M. K. (2003). A validation study of Korea positive and negative affect schedule: the PANAS scales. *Kor. J. Clin. Psychol.* 22 935–946.

[B37] Lee K. Kim J. Yeon B. Kim S. Chae J. (2013). Development and standardization of extended ChaeLee Korean facial expressions of emotions. *Psychiatry Investig.* 10 155–163. 10.4306/pi.2013.10.2.155 PMC3687050 23798964

[B38] Lee S. H. Kim E. Y. Kim S. Bae S. M. (2010). Event-related potential patterns and gender effects underlying facial affect processing in schizophrenia patients. *Neurosci. Res.* 67 172–180. 10.1016/j.neures.2010.03.001 20214929

[B39] Leppänen J. M. (2006). Emotional information processing in mood disorders: a review of behavioral and neuroimaging findings. *Curr. Opin. Psychiatry* 19 34–39. 10.1097/01.yco.0000191500.46411.00 16612176

[B40] Li H. Chan R. C. Zhao Q. Hong X. Gong Q. Y. (2010). Facial emotion perception in Chinese patients with schizophrenia and non-psychotic first-degree relatives. *Prog. Neuropsychopharmacol. Biol. Psychiatry* 34 393–400. 10.1016/j.pnpbp.2010.01.007 20079792

[B41] Lyons M. J. Akamatsu S. Kamachi M. Gyoba J. Budynek J. (1998). “The Japanese female facial expression (JAFFE) database,” in *Proceedings, Third IEEE International Conference on Automatic Face and Gesture Recognition* (Nara: IEEE Computer Society) 200–205. 10.1109/AFGR.1998.670949

[B42] Mandal M. K. Pandey R. Prasad A. B. (1998). Facial expressions of emotions and schizophrenia: a review. *Schizophr. Bull.* 24 399–412. 10.1093/oxfordjournals.schbul.a033335 9718632

[B43] McCarthy G. Puce A. Gore J. C. Allison T. (1997). Face-specific processing in the human fusiform gyrus. *J. Cogn. Neurosci.* 9 605–610. 10.1162/jocn.1997.9.5.605 23965119

[B44] Nicholls M. E. Wolfgang B. J. Clode D. Lindell A. K. (2002). The effect of left and right poses on the expression of facial emotion. *Neuropsychologia* 40 1662–1665. 10.1016/S0028-3932(02)00024-6 11992654

[B45] Niedenthal P. M. Halberstadt J. B. Margolin J. Innes-Ker A. H. (2000). Emotional state and the detection of change in facial expression of emotion. *Eur. J. Soc. Psychol.* 30 211–222. 10.1002/(SICI)1099-0992(200003/04)30:2<211::AID-EJSP988>3.0.CO;2-3

[B46] Öhman A. Lundqvist D. Esteves F. (2001). The face in the crowd revisited: a threat advantage with schematic stimuli. *J. Pers. Soc. Psychol.* 80 381–396. 10.1037/0022-3514.80.3.381 11300573

[B47] Olofsson J. K. Nordin S. Sequeira H. Polich J. (2008). Affective picture processing: an integrative review of ERP findings. *Biol. Psychol.* 77 247–265. 10.1016/j.biopsycho.2007.11.006 18164800 PMC2443061

[B48] Orgeta V. Phillips L. H. (2007). Effects of age and emotional intensity on the recognition of facial emotion. *Exp. Aging Res.* 34 63–79. 10.1080/03610730701762047 18189168

[B49] Pessoa L. McKenna M. Gutierrez E. Ungerleider L. (2002). Neural processing of emotional faces requires attention. *Proc. Natl. Acad. Sci. U.S.A.* 99 11458–11463. 10.1073/pnas.172403899 12177449 PMC123278

[B50] Rocca C. C. D. A. Heuvel E. V. D. Caetano S. C. Lafer B. (2009). Facial emotion recognition in bipolar disorder: a critical review. *Rev. Bras. Psiquiatr.* 31 171–180. 10.1590/S1516-44462009000200015 19578691

[B51] Rotter N. G. Rotter G. S. (1988). Sex differences in the encoding and decoding of negative facial emotions. *J. Nonverb. Behav.* 12 139–148. 10.1007/BF00986931

[B52] Ruffman T. Henry J. D. Livingstone V. Phillips L. H. (2008). A meta-analytic review of emotion recognition and aging: implications for neuropsychological models of aging. *Neurosci. Biobehav. Rev.* 32 863–881. 10.1016/j.neubiorev.2008.01.001 18276008

[B53] Russell J. A. (1994). Is there universal recognition of emotion from facial expressions? A review of the cross-cultural studies. *Psychol. Bull.* 115 102–141. 10.1037/0033-2909.115.1.102 8202574

[B54] Russell J. A. (2003). Core affect and the psychological construction of emotion. *Psychol. Rev.* 110 145–172. 10.1037/0033-295X.110.1.145 12529060

[B55] Saegusa C. Intoy J. Shimojo S. (2015). Visual attractiveness is leaky: the asymmetrical relationship between face and hair. *Front. Psychol.* 6:377 10.3389/fpsyg.2015.00377 PMC4390982 25914656

[B56] Salters-Pedneault K. Roemer L. Tull M. T. Rucker L. Mennin D. S. (2006). Evidence of broad deficits in emotion regulation associated with chronic worry and generalized anxiety disorder. *Cogn. Ther. Res.* 30 469–480. 10.1007/s10608-006-9055-4

[B57] Sheehan D. V. Lecrubier Y. Sheehan K. H. Amorim P. Janavs J. Weiller E. (1998). The mini-international neuropsychiatric interview (M.I.N.I): the development and validation of a structured diagnostic psychiatric interview for DSM-IV and ICD-10. *J. Clin. Psychiatry* 59(Suppl. 20) 22–33. 9881538

[B58] Shih F. Y. Chuang C. F. Wang P. S. (2008). Performance comparisons of facial expression recognition in JAFFE database. *Int. J. Pattern Recogn.* 22 445–459. 10.1142/S0218001408006284

[B59] Stiensmeier-Pelster J. Martini A. Reisenzein R. (1995). The role of surprise in the attribution process. *Cogn. Emot.* 9 5–31. 10.1080/02699939508408963

[B60] Tagai K. Ohtaka H. Nittono H. (2016). Faces with light makeup are better recognized than faces with heavy makeup. *Front. Psychol.* 7:226 10.3389/fpsyg.2016.00226 PMC4771839 26973553

[B61] Watson D. Clark L. A. Tellegen A. (1988). Development and validation of brief measures of positive and negative affect: the PANAS scales. *J. Pers. Soc. Psychol.* 54 1063–1070. 10.1037/0022-3514.54.6.1063 3397865

[B62] Wyer N. A. Hollins T. J. Pahl S. Roper J. (2015). The hows and whys of face memory: level of construal influences the recognition of human faces. *Front. Psychol.* 6:1524 10.3389/fpsyg.2015.01524 PMC4595788 26500586

[B63] Yin L. Wei X. Sun Y. Wang J. Rosato M. J. (2006). “A 3D facial expression database for facial behavior research,” in *Proceedings of the 7th International Conference on Automatic Face and Gesture Recognition (FGR ’06)* (New York, NY: IEEE) 211–216.

[B64] Yoo S. Kim Y. Noh J. Oh K. Kim C. Namkoong K. (2006). Validity of Korean version of the mini-international neuropsychiatric interview. *Anxiety Mood* 2 50–55.

[B65] Yrizarry N. Matsumoto D. Wilson-Cohn C. (1998). American-Japanese differences in multiscalar intensity ratings of universal facial expressions of emotion. *Motiv. Emot.* 22 315–327. 10.1023/A:1021304407227

