# Supplementary material for: Development of the Korean Facial Emotion Stimuli: Korea University Facial Expression Collection 2nd Edition
Source: Front Psychol. 2017 May 12;8:769. doi: 10.3389/fpsyg.2017.00769 (PMC5427125; doi:10.3389/fpsyg.2017.00769)
Supplement: Supplementary file 1 [file Table_1.DOCX]

Supplement Table 1. Correlations between hit rates and emotional state.

|  | Positive Affect | Negative Affect |
| --- | --- | --- |
| Neutral | -.052 | -.148 |
| Surprise | -.146 | -.187 |
| Happiness | .081 | -.123 |
| Negative emotions | -.049 | .022 |
