# Supplementary material for: Development of the Korean Facial Emotion Stimuli: Korea University Facial Expression Collection 2nd Edition
Source: Front Psychol. 2017 May 12;8:769. doi: 10.3389/fpsyg.2017.00769 (PMC5427125; doi:10.3389/fpsyg.2017.00769)
Supplement: Supplementary file 2 [file Table_2.DOCX]

Supplement Table 2. Hit rates for each emotional category on the FaFD, KDEF, ChaeLee-E and KUFEC-II (%)

|  | RaFD | KDEF | 3D FES | ChaeLee-E | WSEFEP | KUFEC-I | KUFEC-II |
| --- | --- | --- | --- | --- | --- | --- | --- |
| Overall | 82 | 71.9 | - | - | 82.4 | - | 80.8 |
| Happiness | 98 | 92.7 | 92 | 95.5 | 87.6 | 99.7 | 98.1 |
| Sadness | 80 | 76.7 | 64 | 89.2 | 86.9 | 91.6 | 85.5 |
| Surprise | 90 | 77.1 | - | 85.5 | 89.0 | 92.3 | 94.3 |
| Fear | 81 | 43.0 | 45 | 49.0 | 68.9 | 12.9 | 44.8 |
| Anger | 85 | 78.8 | 47 | 87.6 | 86.4 | 91.1 | 87.2 |
| Disgust | 81 | 72.2 | 66 | 69.1 | 90.7 | 66.1 | 63.8 |
| Neutral | 83 | 62.6 | 75 | 92.2 | 63.0 | 93.6 | 92.3 |

* RaFD - Radboud Face Database; KDEF- Karolinska Directed Emotional Faces database; 3D FES- 3D Facial Emotional Stimuli; ChaeLee-E – Chae Lee Korean Facial Expressions of Emotions; WSEFEP- Warsaw Set of Emotional Facial Expression Pictures; KUFEC-I- Korea University Facial Expression Collection 1^ST^ Edition; KUFEC-II- Korea University Facial Expression Collection 2^ND^ Edition.
