# Supplementary material for: Development of the Korean Facial Emotion Stimuli: Korea University Facial Expression Collection 2nd Edition
Source: Front Psychol. 2017 May 12;8:769. doi: 10.3389/fpsyg.2017.00769 (PMC5427125; doi:10.3389/fpsyg.2017.00769)
Supplement: Supplementary file 3 [file Table_3.DOCX]

Supplement Table 3. Percentage of chosen emotions per intended emotional expression (%)

|  | Happiness | Sadness | Surprise | Fear | Anger | Disgust | Neutral |
| --- | --- | --- | --- | --- | --- | --- | --- |
| Happiness | 97.11 | 0.45 | 0.49 | 0.33 | 0.68 | 0.61 | 0.33 |
| Sadness | 0.26 | 84.38 | 1.17 | 1.12 | 3.35 | 4.50 | 5.22 |
| Surprise | 0.70 | 1.26 | 92.72 | 3.23 | 0.52 | 0.63 | 0.94 |
| Fear | 1.05 | 5.07 | 26.85 | 49.76 | 0.00 | 16.52 | 0.75 |
| Anger | 0.26 | 2.81 | 0.35 | 1.36 | 87.33 | 7.03 | 0.87 |
| Disgust | 0.45 | 8.81 | 1.34 | 2.16 | 23.58 | 63.46 | 0.21 |
| Neutral | 1.49 | 2.15 | 0.71 | 0.52 | 2.10 | 0.54 | 92.50 |
